# CSI Amplitude Fingerprinting for Indoor Localization with Dictionary Learning

**DOI:** 10.3390/e23091164

**Published:** 2021-09-04

**Authors:** Wen Liu, Xu Wang, Zhongliang Deng

**Affiliations:** School of Electronic Engineering, Beijing University of Posts and Telecommunications, Beijing 100876, China; 384926220@bupt.edu.cn (X.W.); dengzhl@bupt.edu.cn (Z.D.)

**Keywords:** indoor localization, channel state information, dictionary learning, sparse coding

## Abstract

With the rapid growth of the demand for location services in the indoor environment, fingerprint-based indoor positioning has attracted widespread attention due to its high-precision characteristics. This paper proposes a double-layer dictionary learning algorithm based on channel state information (DDLC). The DDLC system includes two stages. In the offline training stage, a two-layer dictionary learning architecture is constructed for the complex conditions of indoor scenes. In the first layer, for the input training data of different regions, multiple sub-dictionaries are generated corresponding to learning, and non-coherent promotion items are added to emphasize the discrimination between sparse coding in different regions. The second-level dictionary learning introduces support vector discriminant items for the fingerprint points inside each region, and uses Max-margin to distinguish different fingerprint points. In the online positioning stage, we first determine the area of the test point based on the reconstruction error, and then use the support vector discriminator to complete the fingerprint matching work. In this experiment, we selected two representative indoor positioning environments, and compared the DDLC with several existing indoor positioning methods. The results show that DDLC can effectively reduce positioning errors, and because the dictionary itself is easy to maintain and update, the characteristic of strong anti-noise ability can be better used in CSI indoor positioning work.

## 1. Introduction

With the rapid development of mobile devices and wireless technologies, research into location-based services (LBSs) has gradually become a popular topic, and it is essential to obtain high-precision location information [1,2]. Unlike outdoor positioning with line-of-sight propagation paths (e.g., the Global Positioning System (GPS)), indoor positioning faces a challenging wireless signal propagation environment [3]. This leads to the influence of multipath, non-line-of-sight (NLOS) and delay distortion in the indoor environment.

In addition to the accuracy requirements, the indoor positioning system should also have low complexity, and the online processing time of mobile devices should be short [3]. Among many wireless indoor positioning technologies, due to the wide deployment of wireless networks and equipment, with low price and other characteristics, WiFi technology has gradually become an ideal choice for indoor positioning. At the same time, fingerprint-based indoor positioning technology has also become an effective method to meet these requirements. Location fingerprints associate a location in the actual environment with a certain “fingerprint”, and a location corresponds to a unique fingerprint; thus, a large amount of data storage space is essential for building a fingerprint library that is convenient for real-time location estimation.

### 1.1. Fingerprinting Localization

Due to its simplicity and low hardware requirements, many existing indoor positioning systems use RSS as a fingerprint [4,5,6]. For example, the Horus system [7] uses probabilistic methods to estimate the position from RSS data. However, due to the multipath effect in the indoor environment, the RSS value usually changes greatly over time. Even for fixed equipment, such high variability will bring about a large positioning error. In addition, in an orthogonal frequency division multiplexing (OFDM) system, the RSS value does not use a large number of subcarriers to obtain richer multipath information. Recently, a popular method has been to obtain channel state information (CSI) from certain WiFi network interface cards (NICs), which can be used as fingerprints to improve indoor positioning performance. For example, the fine-grained indoor fingerprint system (FIFS) [8] uses the weighted average CSI value on multiple antennas to act as a fingerprint. Another example is the DeepFi system [9], which uses 90 sub-carriers collected by three antennas to train a deep network through deep learning to obtain the best weight as a fingerprint.

With the popularization of machine learning applications, K-nearest neighbors (KNNs), neural networks, support vector machines, etc., have been widely used in fingerprint-based indoor positioning. These methods will have some obvious inadaptability when applied to fingerprint positioning. For example, KNN takes the inverse of the Euclidean distance between the observed RSS value and its K nearest training samples as the weight, and then uses the weighted average of the positions of the K training samples to determine the position of the test sample. It can be seen that an obvious limitation of KNN is that it needs to store all RSS training samples, which will represent a significant storage burden. For another example, the support vector machine uses a kernel function to solve the randomness and incompleteness of the RSS value, but it has high computational complexity.

### 1.2. Dictionary Learning

It can be seen that the key step of fingerprint positioning is to extract features from fingerprint point signals and build a fingerprint library with a low storage cost and fast search speed. In recent years, the most popular representation learning methods are deep learning and dictionary learning. Dictionary learning is also called sparse representation or sparse coding. It learns a set of atoms and combines them to form a dictionary, so that an input signal can use it to acquire sparse representation, and use the linear combination of atoms in the dictionary to reconstruct the signals, and the sparse representation coefficient specifies how to select “useful” atoms from the dictionary to participate in signal reconstruction [10]. Compared to deep learning through the complex deep neural network to extract the semantic characteristics [9,11], a significant advantage of sparse representation is that it can reduce the dimension of complex signals and extract features in a simple way, which makes the classification of the follow-up process use a more simple and direct classifier (e.g., linear classifier). At the same time, this simple fingerprint can meet the needs of a quickly building fingerprint database and fingerprint matching. In addition, it is worth noting that sparse induction regularization and sparse representation have proven to be very powerful complex signal representation methods in recent studies. Therefore, we can naturally consider using dictionary learning for indoor fingerprint location.

Existing DL methods applied to the field of representation and classification can be divided into two categories: unsupervised DL and discriminant DL [12,13,14]. Unsupervised DL does not use any prior information (such as class label information) about the training data. The goal of this approach is to train a learning dictionary that can minimize the error of data reconstruction, thus achieving the effect of the compression of storage space and denoising. K-singular value decomposition (KSVD) [15] is one of the most representative DL methods. The main purpose of KSVD is to learn an over-complete dictionary by popularizing k-means, but at the same time, KSVD values are suitable for the efficient representation of training data, rather than for processing classification tasks. In contrast, discriminant DL methods have been shown to be more effective in dealing with classification tasks by introducing the label information of training data into the objective function [16,17,18,19]. Specifically, this supervised approach to generating learning dictionaries can be divided into three broad categories:

1. Dictionary items are selected from an initialized large dictionary to generate a more compact dictionary.
2. The discriminant term is incorporated into the objective function to enhance the identifiability of sparse coding. Among them, distinguishing KSVD (D-KSVD) [20], label consistent KSVD (LC-KSVD) [21] and Fisher discrimination DL (FDDL) [22] are three representative algorithms. D-KSVD introduces the classification discriminant error in the objective function, thereby increasing the classification ability; LC-KSVD further merges the label consistency constraint in the objective function of KSVD to ensure that sparse coding can represent data while also having a high degree of discrimination [12,23]; FDDL looks for structured dictionaries and forces sparse coding to have smaller intra-class walks and larger inter-class walks.
3. The goal of the third category is to calculate category-specific sub-dictionaries, thereby encouraging each sub-dictionary to correspond to a single category. For example, in [24], it introduces an incoherent promotion term to ensure the independence between the learned sub-dictionaries that serve a specific category. Zhou et al. [25] proposed a DL algorithm that associates target categories by learning multiple dictionaries.

### 1.3. Limitations

It is worth noting that most of the aforementioned DL methods still have some known drawbacks. First, in order to obtain sparse codes, most existing methods use l0 or l1 norm sparsity constraints for sparse codes, which leads to time-consuming training. Secondly, when the dictionary learning containing discriminative items is applied to pattern classification, it usually requires a large number of atoms to obtain satisfactory results. Therefore, a relatively large dictionary must be trained, and it will increase the burden of the matching stage. The starting point of a lightweight fingerprint database and fast matching test points is contrary to the starting point. However, if the training method of the class-specific sub-dictionary is adopted, the optimization process will be very time-consuming, especially in the case of a large number of classes.

As we all know, current research shows that dictionary learning and sparse representation have excellent performance in image denoising and classification tasks. However, when faced with the channel state information that contains rich features and is used for indoor fingerprint positioning, whether the dictionary learning still has good adaptability and whether it can accurately complete the fingerprint matching work remains to be proved by experiments.

### 1.4. Contributions

**Figure 1:**
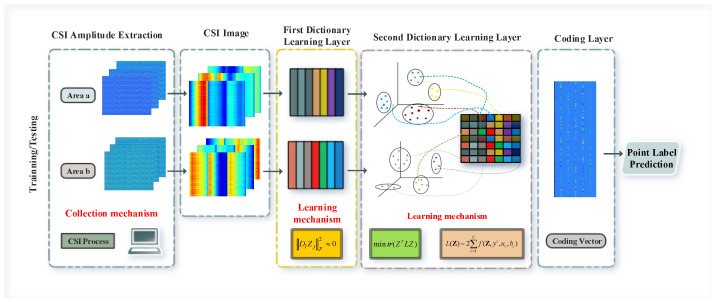
Framework of the proposed method.

In this paper, we propose a two-layer sub-dictionary learning architecture, as shown in Figure 1. First of all, for the CSI amplitude information, we use a ConFi-like method to construct three channels using sub-carriers collected by three antennas to achieve the graphical visualization of CSI so as to better cater to the advantages of dictionary learning in image processing. Subsequently, in first-level dictionary learning, regions are divided as complex indoor environments are where region-specific rather than fingerprint-specific sub-dictionaries are trained, and non-coherent promotion items are introduced to ensure the independence of sub-dictionaries between regions. This means that it will not train a large dictionary with an excessively large scale due to the complexity of the scene, and it can alleviate the optimization problem of sub-dictionaries with too many categories in the optimization class-specific dictionary. At the same time, in order to reduce the computational cost of regional discrimination, we introduce the l2,1 norm instead of the traditional l1 or l2 norm to further reduce the optimization cost. In the second-level dictionary learning, in order to enhance the matching ability of fingerprint points in the region, support vector discriminant items are introduced to promote the discriminative ability of sparse coding. Considering that CSI data often contain abnormal points or outliers, the graphical Laplacian matrix constructed on the original CSI training samples cannot truthfully describe the manifold structure, so we use the atoms in the second-level dictionary’s local constraints. When the dictionary learning process is completed, we will obtain the sub-dictionaries and multi-class support vector machines for each region at the same time, and use them to sparsely encode and classify the test data.

## 2. Preliminary

### 2.1. CSI

OFDM is a digital multi-carrier modulation method with limited bandwidth in wireless communication. Its modulation and demodulation are implemented based on inverse fast Fourier transform (IFFT) and fast Fourier transform (FFT), respectively. OFDM has become the most widely used multi-carrier modulation technology. In WiFi networks, OFDM divides the signal into multiple orthogonal sub-channels with different frequencies. The channel state information reflects the characteristics of the communication link between the transmitter and the receiver, including the effects of distance, scattering, and fading on the signal. Channel state information can be expressed as

(1) $$\vec{Y}=H\cdot \vec{X}+\vec{N}$$

Here, $\vec{X}$ and $\vec{Y}$ represent the transmitting and receiving signal vectors, the vector $\vec{N}$ represents additive white Gaussian noise, and the *H* matrix represents channel state information. It is a collection of channel information for each subcarrier, and it can be estimated using $\vec{X}$ and $\vec{Y}$. Where $H={\left[H_{1},H_{2},...,H_{n}\right]}^{T}$, *n* is the number of subcarriers, and $H_{i}$ appears in the plural form:

(2) $$H_{i}=\left|H_{i}\right|exp\left\{j∠H_{i}\right\}$$

Here, $\left|H_{i}\right|$ and $∠H_{i}$ are the amplitude response and phase response of the subcarrier *i*, respectively. The physical meaning of CSI amplitude is the quantification of the signal power attenuation after multi-path fading [26,27]. The user motion in the WiFi-enabled area affects the wireless signal propagation and changes the amplitude of the signal arriving at the receiver, leading to amplitude variation. Therefore, we can use amplitude as a unique fingerprint feature of the indoor location. In theory, the phase contains more information than the amplitude and can be used to depict the signal changes. However, as phase is periodic compared to amplitude and its measurement value is affected by device clock and carrier frequency, it must be calibrated to generate practical phase features. Furthermore, due to the fading effect and frequency offset, the phase of the CSI is a bit noisy compared to the amplitude, so this article only uses the amplitude information of the CSI. The experimental data in this article will be based on 90 sub-carriers from three antennas.

**Figure 2:**
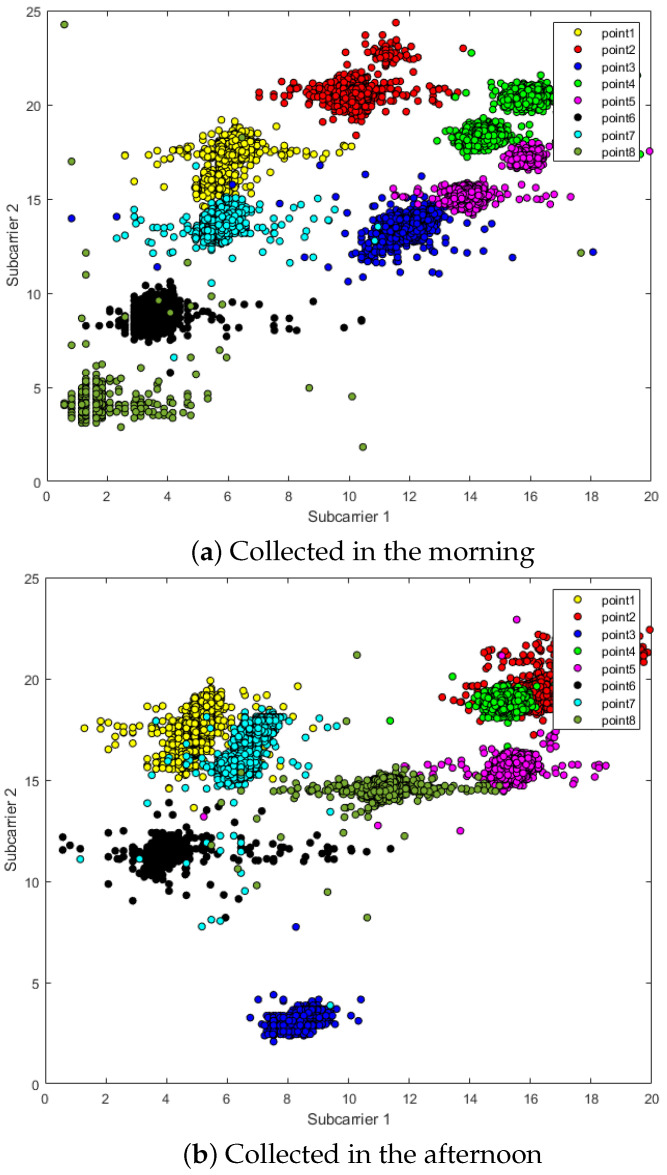
The fluctuation of CSI amplitude: (**a**) CSI amplitude data collected in the morning; and (**b**) CSI amplitude data collected in the afternoon in the same environment.

Since CSI contains more channel characteristics and thus maps richer scene information, this also means that CSI is very sensitive to changes in the environment. In addition to the movement of people, even the movement of objects in the scene will be reflected in the form of numerical fluctuations in the CSI amplitude. Figure 2 shows two sub-carriers in the CSI data, measured twice in the morning and afternoon in a fixed scene. There is almost no item movement in the scene, but the two sub-carriers “point2” and “point8” in the picture still occur with obvious deviation.

### 2.2. Sparse Coding

Sparse representation focuses on signal modeling, and the goal is to obtain a faithful expression of the original signal, which can well separate the signal from meaningless noise. Deep learning focuses more on extracting signal features to serve downstream tasks [28,29]. For CSI amplitude, which is very sensitive to environmental noise, dictionary learning can extract location information more effectively than environmental noise, so it can be applied to fingerprint location.

Sparse coding refers to the process in which the original signal *y* obtains the sparse signal *x* based on a dictionary $D=\left[d_{1},d_{2},...,d_{p}\right]\in R^{m\times p}$, where the dictionary *D* is learned by a dictionary learning algorithm. It usually is expressed as the following constrained optimization goal:

(3) $$\underset{x}{min}{∥x∥}_{0}\;s.t.\;y=Dx$$

Here, ${∥\cdot ∥}_{0}$ is $l_{0}-$ norm, with the goal of calculating the non-zero items of the vector. However, the equality constraint y=Dx is too strict for this optimization problem, so we relax the optimization problem with a smaller threshold. By generalizing the K-means clustering process, Aharon et al. developed the K-SVD algorithm, which can learn an over-complete dictionary most suitable for the given data. The objective function of the K-SVD algorithm is as follows:

(4) $$\underset{D,X}{min}{∥Y-DX∥}_{F}^{2}\;s.t.{∥x_{i}∥}_{0}\leq T_{0}$$

where $T_{0}$ is a given sparsity constraint. Equation (4) can be solved by alternately updating *D* and *X*. However, although K-SVD has excellent performance in the field of image compression and denoising, the algorithm itself is not designed for classification problems. In order to make dictionary learning more suitable for classification problems, there are several popular extensions to traditional sparse coding based on KSVD. Zhang et al. proposed the D-KSVD algorithm by introducing a classification error term into the K-SVD framework:

(5) $$\begin{array}{cc} \; & \underset{D,W,X}{min}{∥Y-DX∥}_{F}^{2}+\beta {∥H-WX∥}_{F}^{2}+\lambda {∥W∥}_{F}^{2}\\ \; & s.t.∥x_{i}∥\leq T_{0} \end{array}$$

where $H=[h_{1},h_{2},...,h_{c}]$ is the label matrix of the training data, and *W* is the parameter of a linear classifier. It can be seen from Formula (5) that D-KSVD can simultaneously learn a dictionary and a simple linear classifier. In order to further expand the discriminative ability of encoding vectors, Cai et al. introduced a multi-class SVM regularization term, which is specifically defined as

(6) $$L\left(X\right)=2\sum_{c=1}^{C}f\left(X,y^{c},u_{c},b_{c}\right)$$

where $u_{c}$ is the normal vector associated with the c-th hyperplane of SVM, $b_{c}$ is the corresponding deviation, and $y^{c}=\left[y_{1}^{c},y_{2}^{c},...,y_{n}^{c}\right]$ is defined as when the class label $y_{i}=c$, $y_{i}=1$, otherwise $y_{i}=-1$. In this way, the discriminant term can be reified as $f\left(X,y,u,b\right)={∥u∥}_{2}^{2}+\theta \sum_{i=1}^{n}l\left(x_{i},y_{i},u,b\right)$, where $l(x_{i},y_{i},u,b)$ is the hinge loss function, and θ is the penalty parameter.

In addition, as mentioned above, most of the current research on dictionary learning uses a shallow (single-layer) network structure, and its main purpose is still to decompose the input data into dense bases and sparse coefficients. However, it is difficult for the shallow network structure to completely extract the intrinsic properties of the input data. In recent studies, it has been proven that a deeper network architecture can be constructed from the dictionary learning method. For example, Mahdizadehaghdam et al. proposed a new model that attempts to learn a deep dictionary structure applied to image classification tasks [30]. Unlike traditional deep neural networks, deep learning dictionaries usually extract feature representations at a patch level, and then rely on the learning dictionary of patch outputs for a global sparse feature representation of input data. The model draws on some of the ideas of CNNs [31,32]. First, it directly learns the dictionary from the image features, and then uses the learned dictionary as a candidate pool to learn the next layer of the dictionary, and the learning dictionary between different layers is connected.

## 3. Materials and Methods

### 3.1. CSI Images

First of all, the overall fingerprint positioning system is divided into two phases: the training phase and testing phase. In the training phase, considering the use of the CSI data trajectory discussed above to enhance the positioning effect, it is necessary to collect the CSI of each predefined reference point (RP) multiple times and store it in a database as a fingerprint: for example, in the RP points *i*, we group *H* times the CSI amplitude measurements from *W* subcarriers to construct a H×W matrix:

(7) $$\tilde{A}\left(l_{i}\right)=\left[\begin{array}{cccc} A_{i}^{11} & A_{i}^{12} & ... & A_{i}^{1W}\\ A_{i}^{21} & A_{i}^{22} & ... & A_{i}^{2W}\\ \vdots & \vdots & \ddots & \vdots \\ A_{i}^{H1} & A_{i}^{H2} & ... & A_{i}^{HW} \end{array}\right]$$

Among them, $A_{i}^{hw}$ is the CSI amplitude value from the *w*th subcarrier in the *h*th measurement of the RP point *i*. Considering the excellent performance of dictionary learning in dealing with image classification tasks, we also consider visualizing CSI data. Here, we mainly apply a simple standardization process to obtain clearer data from the original image. First, we calculate the original average amplitude of each image at each position $l_{i}$:

(8) $$A_{i}=\frac{\sum_{1}^{H}\sum_{1}^{W}A_{1}^{hw}}{H\cdot W}$$

For the location point $l_{i}$, the maximum and minimum values of each row are recorded, respectively, and the standardization result of single pixel (CSI amplitude) in the row of *h*, column of *W*, is as follows:

(9) $${\hat{A}}_{i}^{hw}=\frac{A_{i}^{hw}-Min\left(A_{i}^{hw}\right)}{Max\left(A_{i}^{hw}\right)-Min\left(A_{i}^{hw}\right)}$$

Subsequently, the standardized CSI image is:

(10) $$\tilde{\hat{A}}\left(l_{i}\right)=\left[\begin{array}{cccc} {\hat{A}}_{i}^{11} & {\hat{A}}_{i}^{12} & ... & {\hat{A}}_{i}^{1W}\\ {\hat{A}}_{i}^{21} & {\hat{A}}_{i}^{22} & ... & {\hat{A}}_{i}^{2W}\\ \vdots & \vdots & \ddots & \vdots \\ {\hat{A}}_{i}^{H1} & {\hat{A}}_{i}^{H2} & ... & {\hat{A}}_{i}^{HW} \end{array}\right]$$

Finally, in order to maintain the original power of the image, the CSI image at each position $l_{i}$ uses the previously calculated average amplitude to be standardized again:

(11) $$\tilde{\hat{A}}\left(l_{i}\right)=\tilde{\hat{A}}\left(l_{i}\right)\frac{A_{i}}{A_{max}}$$

### 3.2. First Dictionary Learning Layer

First of all, considering the burden of the matching algorithm brought by the fingerprint library containing a large number of fingerprint points, we first trained the region-specific sub-dictionary (sub-fingerprint library) on the first-layer dictionary learning [33]. In order to make the sparse coding of CSI data of different partitions have a high degree of discrimination, we first introduce the following non-coherent promotion items:

(12) $$f\left(D_{l}\right)={∥D_{l}\bar{X_{l}}∥}_{F}^{2}$$

Here, $D_{l}$ corresponds to the sub-dictionary of area l, and $X_{l}$ is the coding coefficient of the CSI training data $Y_{l}$, which is collected on area l of the corresponding sub-dictionary $D_{l}$. $\bar{X_{l}}$ represents the complementary matrix of $X_{l}$, which excludes $X_{l}$ itself from all training data. Among them, $X_{l}$ and partition *l* are corresponding, which means that $Y_{l}$ can be well represented by $X_{l}$ instead of $X_{j},\left(l\neq j\right)$. As in Formula (13), we hope that ${∥D_{l}X_{j}∥}_{F}^{2}$ should be as small as possible in the optimization process, so as to ensure that $D_{l}Xj$ and $Y_{l}$ are not close, and this point will significantly increase the identification effect of fingerprint points when partitioning.

(13) $$\left\{ \begin{array}{c} D_{l}X_{l}\approx Y_{l}\\ D_{l}X_{j}\approx 0 \end{array}\;,\forall l\neq j\right.$$

At the same time, in order to ensure that $X_{l}$ is as sparse as possible, most of the existing models perform the regularization of the $l_{0}$-norm or $l_{1}$-norm on sparseness, which usually leads to complex computation, and this is obviously not suitable for us to train a simple region classification model. It is worth noting that $l_{2,1}$-norm can ensure that the coefficient rows are sparse [21,22], and the calculation of the $l_{2,1}$-norm is efficient and easy, so we use the $l_{2,1}$-norm constraint to ensure sparsity. Finally, we define the first-level dictionary learning objective function as

(14) $$\begin{array}{cc} \; & \underset{D,Z}{min}\sum_{l=1}^{n}\left\{{∥Y_{l}-D_{l}X_{l}∥}_{F}^{2}+\alpha f\left(D_{l}\right)+\tau {∥X_{l}∥}_{2,1}\right\}\\ \; & s.t.{∥d_{v}∥}_{2}^{2}\leq 1,\;v\in \left\{1,...,K\right\} \end{array}$$

Here, α and τ are constant parameters, ${∥d_{v}∥}_{2}^{2}\leq 1$ are dictionary atomic constraints, so that the first calculation process of the layer dictionary learning remains stable. It is worth noting that, considering that the first layer of sub-dictionary learning is only to discriminate the input CSI data, it does not involve more detailed fingerprint point matching, so no additional classifiers are trained here, but an SRC-like method [34] performs the regional discrimination of training data:

(15) $$identity\left(y_{new}\right)=arg\underset{l}{min}∥y_{new}-D_{l}x_{new,l}∥$$

In other words, $y_{new}$ is partitioned according to its coding coefficients. The specific method is to assign it to the object class that can minimize the reconstruction error.

### 3.3. Second Dictionary Learning Layer

In the first layer, we introduced an incoherent promotion term to ensure the sparse representation ability of CSI data in the corresponding region sub-dictionary. In order to make sparse coding also have the ability to locate fingerprints, we first take the sparse coding of the first layer sub-dictionary as the input of the second layer, and introduce a support vector discriminant item to force the sparse coding from different fingerprint points to use max- separated by margin [24]. At the same time, considering the sensitivity and instability of CSI data, the atoms in the dictionary have been proven to track the manifold structure of the training sample to overcome the influence of noise and outliers [25]. Therefore, we introduce a dictionary atomic local constraint item, which can further enhance the authenticity of the description of the CSI data manifold structure by the second-level dictionary learning. Then, we propose the objective function of the second layer as follows:   

(16) $$\begin{array}{cc} \; & \underset{D^{\left(2\right)},Z,U,b}{min}{∥X-D^{\left(2\right)}Z∥}_{F}^{2}+2\lambda_{2}\sum_{c=1}^{C}f\left(Z,x^{c},u_{c},b_{c}\right)\\ \; & \;\;\;+\lambda_{1}tr\left(Z^{T}LZ\right)\\ \; & s.t.{∥d_{k}^{\left(2\right)}∥}^{2}=1,k\in \left\{1,...,K\right\} \end{array}$$

Here, $\lambda_{1}$ and $\lambda_{2}$ are two constant parameters, and $D^{\left(2\right)}$ represents the second-level dictionary. In this way, the sparse code learned by the first-layer dictionary is input into the second-layer dictionary, and the final fingerprint points are obtained through the classifier parameters *u* and *b*.

### 3.4. Optimization

Then, we will show the optimization process of the objective function in the algorithm. First, in the first dictionary learning layer, we initialize the sub-dictionary $D_{l}$ of each region as a random matrix with the unit *F-norm*, and then alternately minimize the objective function in the following steps:

(1) *Fix* *D* *and Optimize* X,Λ: According to the definition of $l_{2,1}$*-norm* [22], ${∥X∥}_{2,1}=2tr\left(X^{T}\Lambda X\right)$, where Λ Is a diagonal matrix, the elements on the diagonal are $\Lambda_{ii}=1/2{∥X^{i}∥}_{2}$, $X^{i}$ means the *i* line of *X*. By deleting irrelevant items, the objective function of the first dictionary learning layer can be simplified to:

(17) $$X^{*}=\arg\underset{X}{min}\varphi \left(X\right)$$

Among them:

(18) $$\varphi \left(X\right)=\sum_{l=1}^{N}\left\{{∥Y_{l}-D_{l}X_{l}∥}_{F}^{2}+\tau tr\left(X_{l}^{T}\Lambda_{l}X_{l}\right)\right\}$$

For each $\Lambda^{i}\neq 0$, by setting the derivative ∂φ(X)/∂X=0, we can obtain the updated closed-form solution of *X* as shown below:

(19) $$X_{l}^{*}={\left(D_{l}^{T}D_{l}+\tau I+\tau \Lambda_{l}\right)}^{-1}D_{l}^{T}Y_{l}$$

Finally, we can update the diagonal matrix Λ by $\Lambda_{ii}=1/2{∥X^{i}∥}_{2}$.

(2) *Fix* *X* *and optimize* *D*: The update of *D* can be expressed as the following problem:

(20) $$\begin{array}{cc} \; & D^{*}=\arg\underset{D}{min}\sum_{l=1}^{N}{∥Y_{l}-D_{l}X_{l}∥}_{F}^{2}+\alpha {∥D_{l}{\bar{Z}}_{l}∥}_{F}^{2}\\ \; & s.t.{∥d_{v}∥}_{2}^{2}\leq 1,v\in \left\{1,...,K\right\} \end{array}$$

Here, we can use the Lagrangian dual function to solve the above optimization problem, so after derivation we can obtain:

(21) g(η)=infYl−DlXlF2+αDlZ¯l=+∑i=1kηl,i(di2−1)

Here, $\eta_{l,i}$ represents the *i*th equality constraint in the *l*th sub-dictionary Lagrange multiplier. If we construct a diagonal matrix $M_{l}\in R^{k\times k}$, the diagonal elements ${\left(M_{l}\right)}_{ii}=\eta_{i}$, then the above formula can be re-expressed as

(22) $$\begin{array}{cc} L(D_{l},\eta )= & {∥Y_{l}-D_{l}X_{l}∥}_{F}^{2}+\alpha ∥D_{l}{\bar{X}}_{l}∥+tr\left(D_{l}^{T}D_{l}M_{l}\right)\\ \; & -tr\left(M_{l}\right) \end{array}$$

By setting the derivative $\partial L\left(D_{l},\eta \right)/\partial D_{l}=0$, we can obtain the closed-form solution of $D_{l}$ as

(23) $$D_{l}^{*}=Y_{l}X_{l}^{T}{\left(X_{l}X_{l}^{T}+\alpha \bar{X_{l}}\bar{X_{l}^{T}}+M_{l}\right)}^{-1}$$

After passing through the first dictionary learning layer, we input the generated sparse code $X_{l}$ into the second dictionary learning layer, and similarly, the optimization strategy of the second dictionary learning layer is also alternately updated:

(3) *Update* *Z*: First, by fixing other variables, the optimization of *Z* can be carried out by column, which is expressed for each column as:

(24) $$\underset{{}_{}z_{i}}{min}{∥x_{i}-Dz_{i}∥}_{2}^{2}+2\lambda_{2}\Theta \sum_{c=1}^{C}l\left(z_{i},x_{i}^{c},u_{c},b_{c}\right)+\lambda_{1}tr\left(z_{i}^{T}Lz_{i}\right)$$

In order to simplify the optimization process, we use the secondary hinge loss function to approximate the original hinge loss function. The definition of the secondary hinge loss function is:

(25) $$\begin{array}{ccc} \; & l(z_{i},x_{i}^{c},u_{c},b_{c})=\; & \left\{\begin{array}{c} {∥x_{i}^{c}\left(u_{c}^{T}z_{i}+b_{c}\right)-1∥}_{2}^{2},x_{i}^{c}\left(u_{c}^{T}z_{i}+b_{c}\right)-1>0\\ 0,\;\;\;\;\;\;t=1\;\mathrm{or}\;(u_{c}^{T}z_{i}+b_{c})-1\leq 0 \end{array}\right. \end{array}$$

Among them, *t* represents the number of iterations. When t=1, the original optimization problem can be simplified to:

(26) $$\underset{z_{i}}{min}{∥x_{i}-D_{l}z_{i}∥}_{2}^{2}+\lambda_{1}tr\left(z_{i}^{T}Lz_{i}\right)$$

This formula has the following closed-form solution:

(27) $$z_{i}={\left(D^{T}D+\lambda_{1}L\right)}^{-1}D^{T}x_{i}$$

Furthermore, when t≥2, the objective function of (24) can be rewritten as

(28) $$\begin{array}{cc} \underset{z_{i}}{min}{∥x_{i}-D_{l}z_{i}∥}_{2}^{2}+\lambda_{1} & tr(z_{i}^{T}Lz_{i})\\ \; & +2\lambda_{2}\theta \sum_{c\in \phi }{∥x_{i}^{c}\left(u_{c}^{T}z_{i}+b_{c}\right)-1∥}_{2}^{2} \end{array}$$

Here, $\phi =\left\{c\leq |1\leq c\leq C,\;x_{i}^{c}\left(u_{c}^{T}z_{c}+b_{c}-1>0\right)\right\}$, (24) also has the following closed-form solution:

(29) $$Z_{i}={\left(D_{1}\right)}^{-1}D_{2}$$

where, $D_{1}=D^{T}D+\lambda_{1}L+2\lambda_{2}\theta \sum_{c\in \phi }u_{c}u_{c}^{T}$, and $D_{2}=D^{T}x_{i}+2\lambda_{2}\theta \sum_{c\in \phi }u_{c}\left(x_{i}^{c}-b_{c}\right)$.

(4) *Update* *D* and *L*: In order to update *D*, we fix other variables, and then minimize (16), then:

(30) $$\underset{D}{min}{∥X-DZ∥}_{F}^{2},\;s.t.{∥d_{k}∥}^{2}=1,k\in \left\{1,...,K\right\}$$

It can be seen that the above formula has become a least squares problem with quadratic constraints. Here, we use the Lagrangian dual function similarly to solving the problem. The Lagrangian dual function can be expressed as

(31) $$g\left(\delta \right)=\underset{D}{inf}\left({∥X-DZ∥}_{F}^{2}+\sum_{k=1}^{K}\delta_{k}\left({∥d_{k}∥}^{2}-1\right)\right)$$

Similarly, we also construct a diagonal matrix Δ here, with elements $\Delta_{kk}=\delta_{k}$ on the diagonal, and then (20) can be transformed into:

(32) $$L\left(D,\delta \right)={∥X-DZ∥}_{F}^{2}+tr\left(D^{T}D\Delta \right)-tr\left(\Delta \right)$$

In the same way, setting the first derivative to 0, we can obtain the closed-form solution of *D* as

(33) $$D=XZ^{T}{\left(ZZ^{T}+\Delta \right)}^{-1}$$

When *D* is updated, we update the graph Laplacian matrix *L* through (24).

(5) *Update* *U* and *b*: Fixed other variables, (16) about *U* and *b* is summarized as the following questions:

(34) $$\underset{U,b}{min}\sum_{c=1}^{C}\left\{{∥u_{c}∥}_{2}^{2}+\theta \sum_{i=1}^{n}l\left(z_{i},x_{i}^{c},u_{c},b_{c}\right)\right\}$$

The above formula is a multi-class linear SVM problem, which can be solved by the SVM solver proposed by [21]. Finally, the optimization process of our proposed algorithm is shown in Algorithm 1. Since the objective function proposed in (16) is non-convex, the algorithm cannot converge to the global minimum. However, as the objective function decreases, we can finally obtain a satisfactory solution.
**Algorithm 1** Optimization Procedure.**Require:** Training data *Y*, dictionary size *K*, parameters $\alpha ,\tau ,\lambda_{1},\lambda_{2},\theta$
 **while** First DL not converged **do**
  t=t+1
  **for**
*l* = 1 to *L*
**do**
   Update the sparse codes $X_{l}^{\left(t\right)}$ by (19)
  **end for**
  $\Lambda_{ii}=1/2{∥X^{i}∥}_{2}$
  Update the dictionary $D_{l}^{\left(t\right)}$ by (23)
  Initialize U,b
  **while** Second DL not converged **do**
   **for**
l=1 to *L* **do**
    Construct the graph Laplacian matrix $L_{l}$
    Update $D_{l}^{\left(2\right)}$ by (33)
    **for**
i=1 to *n* **do**
     Update $z_{l,i}$ by using (29)
     **for**
c=1 to *C* **do**
      Update $U_{l,c}$ and $b_{l,c}$ by solving (34)     **end for**
    **end for**
   **end for**
  **end while**
 **end while**
 **return** Dictionary *D*,$D^{\left(2\right)}$, Parameters: U,b


## 4. Experiments

In this section, we selected two representative indoor scenes, and conducted a lot of experiments on these two scenes to evaluate the effectiveness of the proposed DDLC, including the execution effect of the positioning task; the model is responding to the impact of the robustness and parameter sensitivity in environmental fluctuations of the model.

### 4.1. Experimental Scene Setting

#### 4.1.1. Single Laboratory

**Figure 3:**
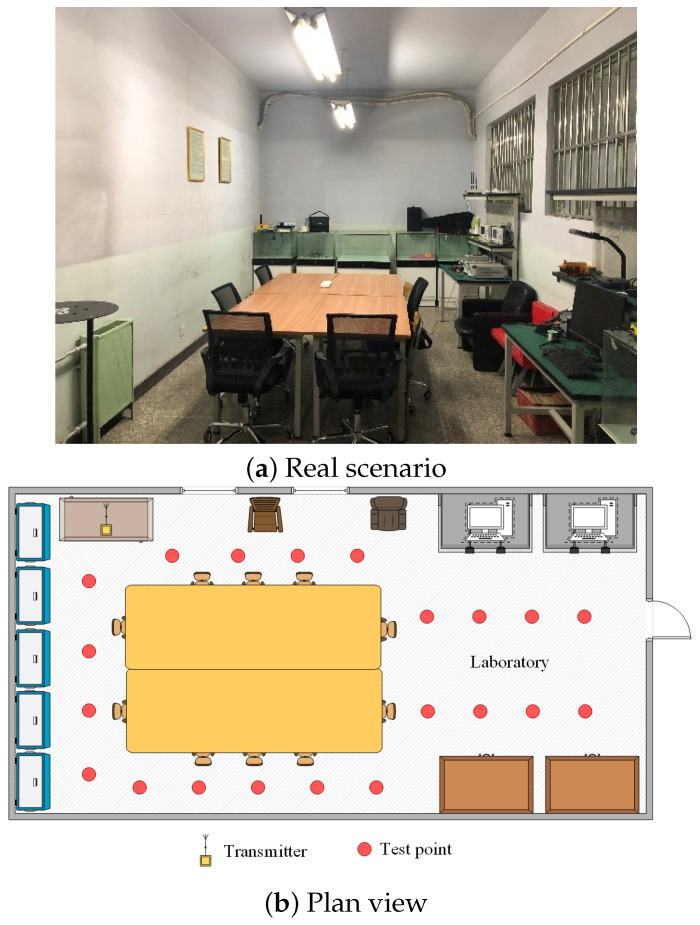
Layout of the laboratory for test positions.

First, we selected a standard laboratory with an experimental area of $19.8\;m^{2}$, for which the layout is shown in Figure 3. This is a common indoor environment, and its center was almost occupied by a large conference table, only containing a small number of personnel and experimental equipment movement, so it was very suitable to verify the ability of our algorithm to deal with CSI data migration. A total of 20 reference points (red) and 1 emitter (yellow) were arranged in the scene. The distance between two adjacent reference points is 0.5m.

#### 4.1.2. Comprehensive Environment

**Figure 4:**
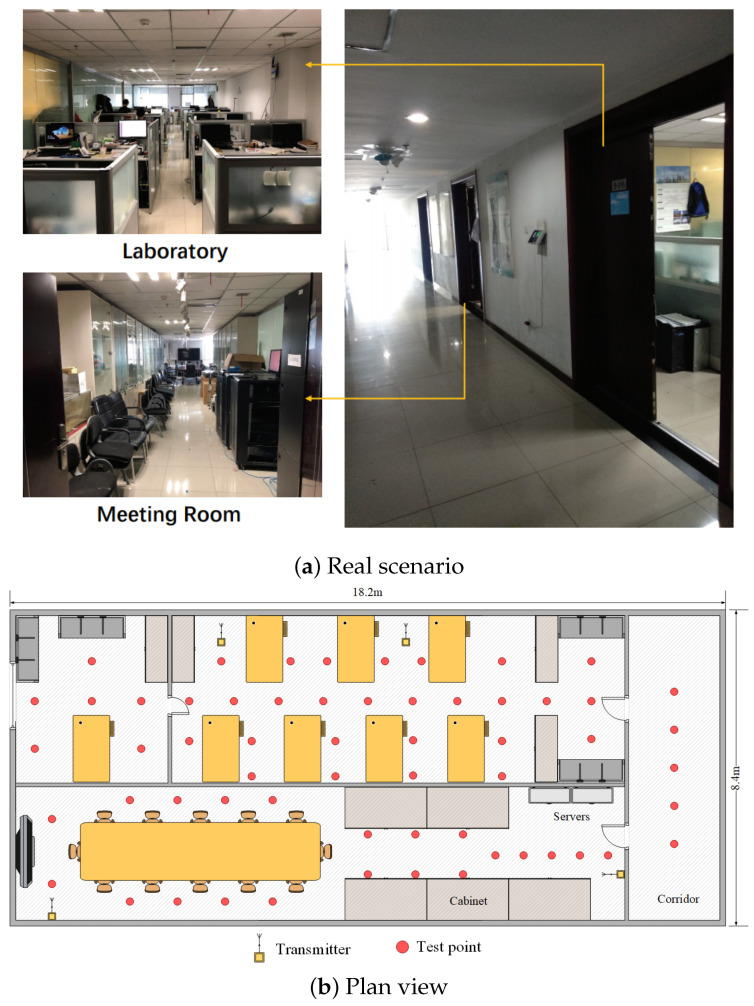
Layout of comprehensive environment for test positions.

As shown in Figure 4, we also selected a composite scene with three typical miscellaneous indoor environments. The total area of the environment is 152.9 $m^{2}$, and the specification of the laboratory is 16.4 × 4.4 $m^{2}$; the specification of the conference room is 16.4 × 4 $m^{2}$; and the specification of the corridor is 8.4 × 1.8 $m^{2}$. There are many desks and computers in the scene, and a glass wall separates the laboratory from the conference room. There are 59 reference points (red) and 4 emitters (yellow) in the scene. The distance between two adjacent reference points is 1.2 m.

### 4.2. Convergence Analysis

**Figure 5:**
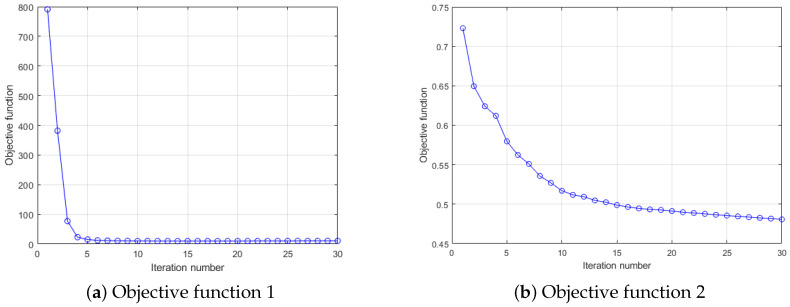
Convergence curve of DDLC for the comprehensive environment.

First of all, in this experiment, we will provide the convergence results of two dictionary learning layers. Here, we mainly analyze the convergence performance of the algorithm through the objective function value. Using the composite scene as shown in Figure 3 and Figure 4, a CSI image is constructed for every 100 consecutive timestamps on 59 reference points, and 10 images are constructed for each reference point for training. Here, for convenience, the number of dictionary atoms is set as the number of training samples. For parameters and other conditions, we refer to the settings of [21,22]. Figure 5 shows the convergence of each objective function when the number of iterations is 30. Notice with two consecutive targets, when the difference of function values is less than 0.001, the iteration stops.

As can be seen from Figure 5, the objective function value of our DDLC does not increase during the iteration process, and more importantly, it will eventually converge to a fixed value. In addition, the first level dictionary tends to be stable in the fifth iteration, while the second level dictionary tends to be stable in approximately 20 iterations, which means that our convergence speed is relatively fast.

### 4.3. Parameter Setting

CSI localization algorithms based on deep neural networks often have many hyperparameters, which require tedious adjustments and cross-validation to obtain better results. Unlike them, our proposed method only has a few parameters to be adjusted. First, in the first-level dictionary learning, there are three adjustable parameters, which are the constant parameters α and τ in the objective function, and the number of atoms in the sub-dictionary *p*.

In this study, we first fixed the number of atoms in the sub-dictionary *p*, and used grid search to determine the two constant parameters of the objective function. For each parameter, we averaged the results based on ten random splits of training and testing data with varied parameters from {$1\times 10^{-3}$, $5\times 10^{-3}$, $1\times 10^{-2}$, $5\times 10^{-2}$, $1\times 10^{-1}$, $5\times 10^{-1}$, 1, 5}.

**Figure 6:**
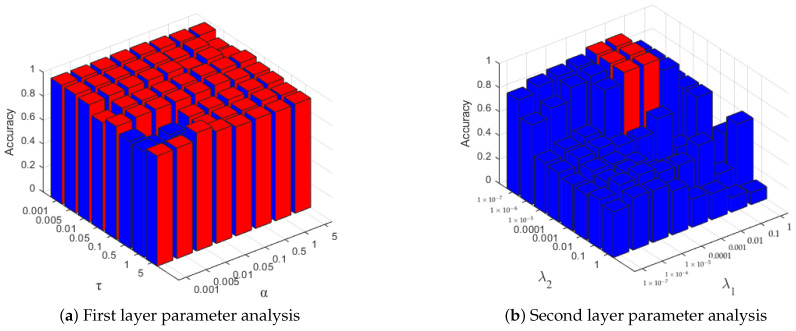
Parameter sensitivity analysis of DDLC.

The result of parameter selection is shown in Figure 6, where the parameter grid with a partition accuracy rate of less than 95% is indicated in blue, and the parameter grid with a partition accuracy rate of more than 95% is indicated in red. We can find that our proposed algorithm performs well in a wide range of parameter selections in each group, which means that DDLC is insensitive to the model parameters.

### 4.4. Positioning Accuacy Assessment

**Figure 7:**
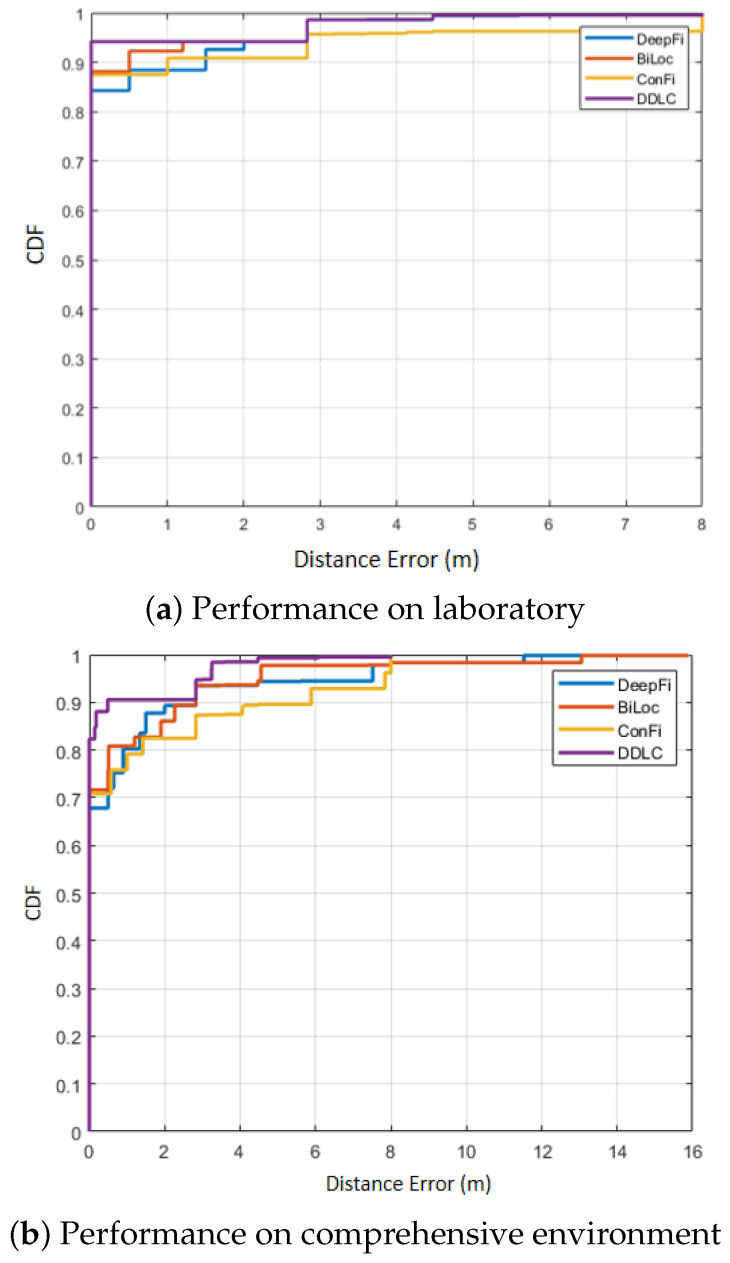
Comparison of CDFs with other positioning methods.

To validate the effectiveness of DDLC, the experiment compares DDLC with three methods which have been verified to perform well in CSI localization according to the existing literature. The three methods are DeepFi [9], BiLoc [18] and ConFi [35]. In this experiment, Table 1 and Figure 7 present the mean errors, standard deviations and CDF curves of localization errors for the different methods.

In Table 1, the mean error of DDLC is smallest, which is 0.56 m in the laboratory environment and 0.68 m in the comprehensive environment. From CDF in Figure 7, taking the area under the curve (AUC) as the index, the curve of DDLC performs best compared with other methods. Compared with ConFi, DDLC achieves a 21.1% in for the mean error in the laboratory environment and 36.4% in the comprehensive environment.

The experiments verify that DDLC outperforms other methods in high precision positioning. It can effectively filter the CSI signal and achieve high-precision matching, and the effect is better than the current advanced algorithm.

### 4.5. Comparison of DL Methods

In order to further verify the adaptability of our algorithm in the CSI location problem, we select some classical dictionary learning algorithms to perform comparisons with, including SRC, KSVD, D-KSVD and LC-KSVD. For each DL algorithm, we carefully selected the parameters with reference to the original.

#### 4.5.1. Matching Results on Laboratory

We first tested the fingerprint matching accuracy of each algorithm in the laboratory scene. The scene has 20 fingerprint points, each fingerprint point has 3000 CSI data of time stamp, and every 100 CSI amplitude of time stamp constructs a CSI image.

Six groups were collected by time. We take the first five groups as the training set, the training dictionary, and the last group as the test set. In terms of parameter selection, since the first level dictionary does not play a practical role in the scene.

**Figure 8:**
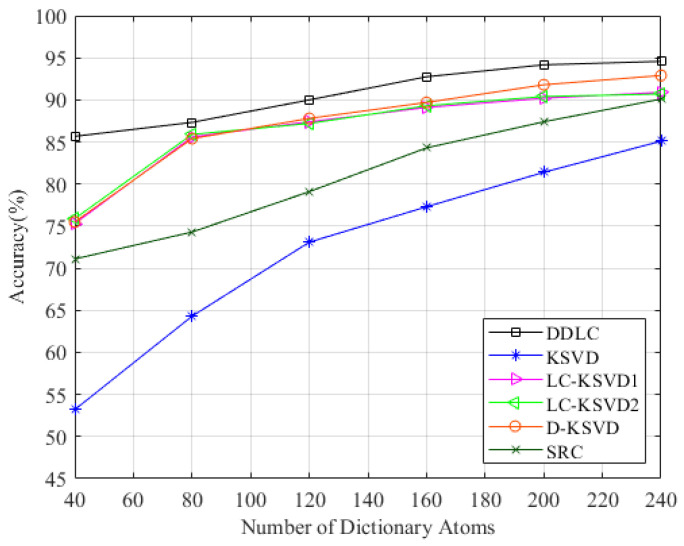
Performance on laboratory with varying dictionary size.

We changed the dictionary specification *k*, such as k=40,80,120,160,200,240, and the result is shown in Figure 8. It can be seen that our method has higher recognition accuracy compared with competitors when the dictionary specification is small.

At the same time, in order to compare the best performance of these DL algorithms, we set the number of dictionary atoms to the maximum (200). For the five measurement data in the laboratory, we input 5, 10, 15, 20, 25 and 30 CSI images for each fingerprint point for training, and the results are shown in Table 2. From the results, we can find that because the laboratory scenario is too simple, and the first dictionary learning layer does not play a practical role, in most cases, our method is slightly better than the competitive method, but the advantage is not obvious. Especially in the case of best performance, DDLC is only 1.7% higher than the traditional D-KSVD, but when the input training image is less, the performance of DDLC is still more outstanding.

#### 4.5.2. Matching Results on Comprehensive Environment

**Figure 9:**
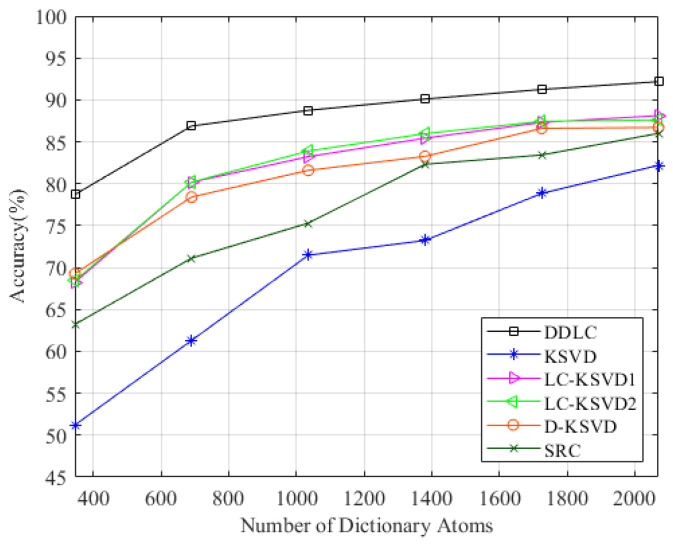
Performance on the comprehensive environment with varying dictionary size.

In order to give full play to the performance of the algorithm, we also performed comparative experiments in the comprehensive environment. The composite scene consists of three regions—a total of 69 fingerprint points. Each fingerprint point still constitutes the CSI amplitude data of 3000 timestamps, and two groups were collected during different time periods. In terms of parameter selection, the parameters of the first level dictionary learning are set as α=1,τ=0.01, and the parameters of the second level dictionary learning are set as $/lambda_{1}=0.01,\lambda_{2}=1\times 10^{-6}$. The dictionary specification is changed from K to k=345,690,1035,1380,1725,2070, respectively. The results are shown in Figure 9. It can be observed that the DDLC is superior to other methods in most cases.

As can be seen from Table 3, when the scene is relatively complex, the two-tier dictionary of DDLC can give full play to its performance, which is obviously better than the classical dictionary learning algorithm in most cases.

## 5. Conclusions

This paper presents a two-layer dictionary learning algorithm for indoor location based on the CSI amplitude feature. First, the CSI data were collected at the preset RP points, and then the CSI amplitude features of the three antennas are imaged as training sets. Secondly, this paper proposes a two-layer dictionary structure algorithm DDLC based on CSI amplitude features. In the first layer of DDLC, an incoherent promotion term is introduced to encourage the sparse coding of CSI amplitude features from different partitions to have high discrimination. Then, the support vector discriminant term is introduced in the second layer of dictionary learning, so that the CSI sparse coding of different reference points can have a discriminant ability, and thus achieve a light weight. The method can realize the preprocessing and fingerprint location of CSI, and has strong generalization ability. In order to further improve the positioning accuracy, we optimized the grid parameters to determine the specific settings of the parameters in the algorithm, and then compared DDLC with the mainstream CSI indoor positioning algorithm and dictionary learning method. The results show that DDLC has significant performance in localization, and it has better adaptability for CSI indoor localization than the classical dictionary learning algorithm. DDLC effectively uses dictionary learning to denoise CSI data, and at the same time, makes its sparse coding with high discrimination, which is more conducive to fingerprint location, and has guiding significance for algorithm design in the field of location.

However, this paper only uses the amplitude characteristics of the CSI signal, and does not carry out experiments in large and complex scenes. In the future, it will further combine the phase information of CSI and carry out experiments in richer scenes.

## Figures and Tables

**Table 1:** Comparison of localization effect with other methods.

Method	Lab. Mean (m)	Lab. Std (m)	Com. Mean (m)	Com. Std (m)
DeepFi	0.78	0.91	1.18	1.75
BiLoc	0.82	1.03	1.29	1.98
ConFi	0.71	0.84	1.07	1.24
DDLC	0.56	0.71	0.68	1.54

**Table 2:** Matching accuracy on the laboratory environment, bold indicates that it is obviously better than other methods.

Num. of Tr. Samp.	10	15	20	25	30
SRC	70.7	79.6	82.2	85.4	90.1
KSVD	61.1	70.7	77.2	82.7	85.1
D-KSVD	63.9	75.8	82.7	87.1	**92.9**
LC-KSVD1	64.7	77.9	82.8	86.7	90.4
LC-KSVD2	64.1	78.3	82.7	87.6	90.7
Our DDLC	**74.4**	**82.6**	**87.8**	**93.3**	**94.6**

**Table 3:** Matching accuracy on the comprehensive environment, bold indicates that it is obviously better than other methods.

Num. of Tr. Samp.	10	15	20	25	30
SRC	67.2	73.4	78.5	84.1	86.0
KSVD	58.0	67.4	76.6	79.3	82.2
D-KSVD	59.8	70.0	78.3	84.3	86.7
LC-KSVD1	60.7	69.9	77.3	82.7	88.1
LC-KSVD2	61.6	71.3	77.8	82.2	87.6
Our DDLC	**81.4**	**87.5**	**89.8**	**91.1**	**92.2**

## Data Availability

Not applicable.
